# Oxoperoxovanadium Complexes of Hetero Ligands: X-Ray Crystal Structure, Density Functional Theory, and Investigations on DNA/BSA Interactions, Cytotoxic, and Molecular Docking Studies

**DOI:** 10.1155/2022/8696420

**Published:** 2022-08-17

**Authors:** Saraswathi Kothandan, Krishnan Thirumoorthy, Antonio Rodríguez-Diéguez, Angappan Sheela

**Affiliations:** ^1^Department of Chemistry, School of Advanced Sciences, Vellore Institute of Technology, Vellore-632014, India; ^2^Department of Inorganic Chemistry, Faculty of Science, University of Granada, Av/Severo Ochoa s/n, Granada 18071, Spain

## Abstract

Oxoperoxovanadium (V) complexes [VO (O)_2_ (nf) (bp)] (**1**) and [VO (O)_2_ (ox) (bp)] (**2**) based on 5-nitro-2-furoic acid (nf), oxine (ox) and 2, 2′ bipyridine (bp) bidentate ligands have been synthesized and characterized by FT-IR, UV-visible, mass, and NMR spectroscopic techniques. The structure of complex 2 shows distorted pentagonal-bipyramidal geometry, as confirmed by a single-crystal XRD diffraction study. The interactions of complexes with bovine serum albumin (BSA) and calf thymus DNA (CT-DNA) are investigated using UV-visible and fluorescence spectroscopic techniques. It has been observed that CT-DNA interacts with complexes through groove binding mode and the binding constants for complexes **1** and **2** are 8.7 × 10^3^ M^−1^ and 8.6 × 10^3^ M^−1^, respectively, and BSA quenching constants for complexes **1** and **2** are 0.0628 × 10^6^ M^−1^ and 0.0163 × 10^6^ M^−1^, respectively. The ability of complexes to cleave DNA is investigated using the gel electrophoresis method with pBR322 plasmid DNA. Furthermore, the cytotoxic effect of the complexes is evaluated against the HeLa cell line using an MTT assay. The complexes are subjected to density functional theory calculations to gain insight into their molecular geometries and are in accordance with the results of docking studies. Furthermore, based on molecular docking studies, the intermolecular interactions responsible for the stronger binding affinities between metal complexes and DNA are discussed.

## 1. Introduction

DNA interaction studies are the active research area at the interface of biology and chemistry and play a key role in the development of new anticancer medicines [1–3]. Small molecules bind through covalent and noncovalent modes of binding and bring about hydrolytic, oxidative, and photolytic cleavage of DNA. It interferes with the replication and transcription processes responsible for cell death [4]. Serum albumin is the main protein in the blood plasma and it plays a significant role in drug pharmacokinetics and pharmacodynamics [5, 6]. Because of its capacity to bind reversibly to a wide range of molecules, it is considered the major transporter of regulatory mediators, metabolic products, nutrients, and fatty acids. Through hydrogen bonding, hydrophobic, and electrostatic interactions, serum albumin neutralizes endogenous and external poisons [7, 8]. Several metal complexes are known to exert their anticancer action through effective binding with nucleases and proteins. In this context, vanadium complexes have been thoroughly studied for their binding efficacies with DNA and BSA. Vanadium forms complexes with many organic molecules and is advantageous over vanadium salts with lesser toxic effects and exerts greater biological efficacy at a very low dosage level. In bioinorganic chemistry, their compounds play a significant role in several enzyme-related biochemical responses like stimulating the functions of myosine ATPase, adenylate kinase, choline esterase, dynein, and phosphofructokinase and also inhibiting the functions of tyrosine phosphatase, glycogen synthase, lipoprotein lipase, and adenylate cyclase [9–13]. The inhibition of phosphatase enzymes instigated the interest in understanding the toxicity profile of vanadium in humans [14]. Ligands containing heteroatoms bind to vanadium and form diverse natures of vanadium species including peroxovanadate, polyoxovanadate monooxo, dioxo, and oxoperoxovanadium species [10].

Among these, peroxovanadium complexes represent an important class of compounds extensively explored for their wide spectrum of promising biological functions including antidiabetic, antitumor, and catalytic activities. They also play an inhibitory role in the hydrolysis of phosphoproteins [15–19]. Oxoperoxovanadium (V) complexes mimic the catalytic action of vanadate-dependent haloperoxidases in oxidizing the halides in the presence of hydrogen peroxide. It also catalyzes the oxidation of hydrocarbons and organic sulfides [20–22]. Besides, peroxovanadium complexes also act as catalysts for many other organic reactions such as oxidation of methyl benzenes, alkenes, tertiary amines, thioanisole, alcohols, and olefin epoxidations [23–25]. Recently, quite a few studies are focused on their potential anticancer activity. These compounds exert their action by generating ROS, interfering in cell cycle arrest, and inhibiting the enzymatic functions [26–28]. The redox behavior of vanadium (V) compounds in biological systems is quite interesting. These compounds are reduced in the biological systems either enzymatically or nonenzymatically and capable of getting reoxidized by O_2_ generating superoxide and peroxovanadyl moieties causing damage to cell organelles causing apoptosis [29].

In this work, we have designed two peroxovanadium (V) complexes based on two heteroligands, such as, 8-hydroxyquinoline (oxine) (ox) and 5-nitro-2-furoic acid (nf) ligands with 2, 2′ bipyridine (bp) ancillary ligand. The ligands, by themselves, possess excellent cytotoxic and antitumor properties, apart from other applications [30–34]. Hence, it is expected that the metal and the ligand exert their action synergistically and bring about good therapeutic efficacy. The complexes are assessed for their DNA and BSA binding potentials in addition to the evaluation of their cytotoxic effect on HeLa cell lines. The geometry, bonding characteristics, and binding propensity with DNA of the metal complexes are evidenced through theoretical calculations and molecular docking studies.

## 2. Experimental

### 2.1. Materials

All the compounds were obtained commercially and utilized as such. Ammonium metavanadate, 2, 2′ bipyridine, oxine, 5-nitro-2-furoic acid, and hydrogen peroxide were obtained from Sigma Aldrich; ethanol, methanol, and acetonitrile solvents in AR grade were obtained from SD Fine Chemicals, and ethidium bromide (EtBr), (Tris (hydroxymethyl) methylamine) HCl, BSA, calf thymus DNA (CT-DNA), Tris-boric acid ethylenediaminetetraacetic acid (TBE) buffer, pBR322, agarose, bromophenol blue, and phosphate buffer saline (PBS) from Merck Millipore were procured.

### 2.2. Instrumentation

A JASCO UV-VIS-NIR V-670 spectrophotometer was used to record electronic spectra. The Hitachi F-7000 FL spectrophotometer was used to record the fluorescence spectra. On a Shimadzu IR affinity-1CE model with resolution IV, FT-IR spectra were obtained using KBr pellets. The Waters Xevo G2-XS-QT high-resolution mass spectrometer was used to record mass spectra (HRMS). The Bruker (400 MHz) spectrometer was used to record NMR spectra with DMSO-d_6_ solvent.

### 2.3. Syntheses of the Complexes **1** [VO (O_2_) (nf) (bp)] and **2** [VO (O_2_) (ox) (bp)]

#### 2.3.1. Preparation of Complex **1**

1 mmol NH_4_VO_3_ was dissolved in 10 ml H_2_O; then, we added, 1 ml of 30% H_2_O_2_. At the same time, we added the methanolic solution of 1 mmol of 5-nitro-2-furoic acid (nf) and 1 mmol of 2, 2′ bipyridine (bp) to the reaction mixture. After 3 h of stirring at room temperature, it was filtered, and on slow evaporation, yielded orange precipitate after 7 days. [VO (O_2_) (nf) (bp)]: molecular formula: [C_15_H_10_N_3_O_8_V]; yield: 80%; orange solid; m.p: 253°C; solubility: ethanol, methanol, acetonitrile, DMSO, and DMF; UV-Vis (acetonitrile): *λ*_max_, nm 239, 279, 382; FT-IR (KBr): *ʋ*, cm^−1^ 947 (V=O), 767 (O-O), 1598 (C=O), 1436 (C-N), 433 (M-N), 524 (M-O); ESI-MS *m/z* = 411 [M]^+^.

#### 2.3.2. Preparation of Complex **2**

**Scheme 1:**
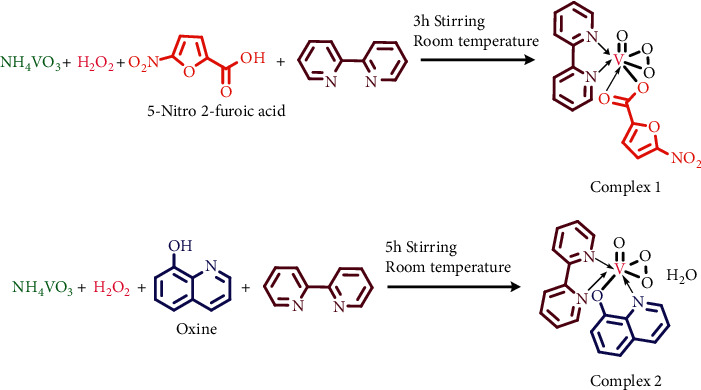
Synthesis of oxoperoxovanadium (V) complexes.

1 mmol NH_4_VO_3_ was dissolved in 10 ml H_2_O and then to it was added 1 ml of 30% H_2_O_2_. At the same time, we added the methanolic solution of 1 mmol of oxine (ox) and 1 mmol of 2, 2′ bipyridine to the reaction mixture. After 5 h of stirring at room temperature, the solution was filtered and on slow evaporation yielded orange crystals of X-ray grade. The syntheses of both the complexes are shown in Scheme 1. [VO (O_2_) (ox) (bp)]: molecular formula: [C_19_H_16_N_3_O_5_V]; yield: 90%; orange solid; m.p: 135°C; solubility: ethanol, methanol, acetonitrile, DMSO, and DMF; UV-Vis (acetonitrile): *λ*_max_, nm 229, 301, 364; FT-IR (KBr): *ʋ*, cm^−1^ 937 (V=O), 837 (O-O), 1282 (C-O), 1463 (C-N), 484 (M-N), 540 (M-O); and ESI-MS *m/z* = 417.06 [M]^+^.

### 2.4. X-Ray Structure Determination

The data collection for the compound **2** crystal was done on a Bruker D8 Venture equipped with a photon detector and graphite monochromated CuK*α* radiation (*λ* = 1.54178 Å). The APEX2 program was used to reduce the data, and SADABS was used to compensate for absorption [35, 36]. The crystal structures were solved using direct techniques in the SIR97 software and improved using full-matrix least-squares on F2 with anisotropic displacement parameters in the WINGX crystallographic tool [37, 38]. Anisotropic temperature factors were applied to all atoms except hydrogen atoms, which are riding their parent atoms with an anisotropic temperature factor arbitrarily set to be 1.2 times that of the corresponding parent.  Table 1 provides the final R (F), wR (F2), and goodness of fit agreement factors, as well as information on data collection and analysis. The crystallographic data for the structure presented in this work (excluding structural factors) have been deposited with the Cambridge Crystallographic Data Centre as supplemental publication nos. CCDC 1950180 for compound **2**. Copies of the data are available for free upon request to the Director, CCDC, 12 Union Road, Cambridge, CB2 1EZ, U.K (Fax: +44-1223-335033; e-mail: deposit@ccdc.cam.ac.uk).

### 2.5. DNA Interaction Studies

For all the studies, stock solutions have been prepared by diluting water: buffer 10 mM (Tris (hydroxymethyl) methylamine) HCl in a ratio of 1 : 10 at pH 7.2. The UV absorbance ratio (A260/A280) of commercial calf thymus DNA in a buffer is roughly 1.9 : 1, showing that the DNA is adequately free of protein. The molar extinction coefficient of 6600 M^−1^ cm^−1^ at *λ*_max_ 260 nm is used to calculate the concentration of DNA. The electronic spectra of the compounds (20 *µ*M) in the presence of increasing CT-DNA concentrations (0–10 *µ*l) were monitored for absorption spectral titration. The fluorescence experiment was carried out with an EtBr bound DNA reference solution and a complex concentration ranging from 0 to 100 *µ*M. Viscosity experiments were carried out in an Ostwald viscometer at room temperature. The flow time was noted and replicated three times. DNA concentration (100 *µ*M) was kept constant and varied complex concentration (20–200 *µ*M). The viscosity values were calculated using the following formula.

(1) $$\begin{array}{c} \eta =\frac{t-t_{0}}{t_{0}}, \end{array}$$

where *t*_0_ represents the buffer alone flows time and *t* represents the DNA containing solution flow time. A graph was plotted (*η*/*η*_0_)^1/3^ versus (complex)/(DNA), where *η*_0_ is the viscosity of DNA-complex and *η* is the viscosity of CT-DNA. The pBR322 plasmid was used in the agarose gel electrophoresis method. The samples were incubated for 2 h at 37°C. The samples with bromophenol blue were loaded onto the ethidium bromide-containing gel and run for 2 h, in 1X TBE buffer (pH 8) at 50 V. The DNA-complex cleavage bands were seen under the UV illuminator of the gel documentation system [39, 40].

### 2.6. BSA Interaction Studies

#### 2.6.1. Fluorescence Quenching Studies

A tryptophan emission quenching study was used to assess the interaction of complexes with BSA (bovine serum albumin). The concentration of BSA (2 *μ*M) in PBS buffer was held constant, while the complex concentration (0–30 *μ*M) increased at room temperature, and the quenching of emission signals at 345 nm (*λ*_ex_ = 290 nm) was measured [41].

#### 2.6.2. UV-Visible Absorption Studies

The absorption titration was carried out with an increasing amount of the complex concentration (0–10 *µ*l) and the BSA (1 *µ*M) concentration was kept constant.

### 2.7. DFT Study

The quantum chemical calculations were carried out for complexes **1** and **2** by utilizing the density functional Theory (DFT). The wB97XD hybrid functional in DFT was used for geometry optimization [42, 43]. The 6–311++g (2d, 2p), split valence basis set, is combined with wB97XD hybrid functional for all the computational calculations [44, 45]. The Gaussian 16 program was used for quantum chemical calculations [46].

### 2.8. Molecular Docking Study

Molecular docking studies on synthetic metal complexes were carried out to get a thorough knowledge of the binding and orientation of metal complexes with DNA [47, 48]. For docking investigations, the X-ray structure of E2 binding DNA (PDB ID:423D) provided by the Brookhaven Protein Data Bank was used [49]. By eliminating the water molecules and magnesium ions, the crystal structure was refined. Gasteiger-Marsili charges [50] hydrogen atoms were added by using AutoDockTools-1.5.6 [51]. AutoDock 4.2 was used for docking calculations, and AutoGrid was used to construct grid potential mappings between the DNA and different atom types. For docking calculations using default settings, a stochastic Lamarckian genetic algorithm approach was applied. During docking computations, AutoDock evaluates conformations using the AMBER force field. The binding energy is calculated using the following scoring function.

(2) $$\begin{array}{c} \Delta G=\Delta G_{\text{vdw}}+\Delta G_{\text{hbond}}+\Delta G_{\text{elec}}+\Delta G_{\text{tor}}+\Delta G_{\text{desolv}}, \end{array}$$

vdw stands for Van der Waal's, hbond for the hydrogen bonding, elec for the electrostatics (elec), Δ*G*_tor_ for the rotation and translation, and Δ*G*_desolv_ stands for the desolvation upon binding and the hydrophobic effect.

### 2.9. In Vitro Cytotoxic Activity

To measure cell viability, the synthesized complexes were tested for cytotoxicity against cervical cancer cells—HeLa cell line—using the MTT method. The cell line was plated individually in 96-well plates at a concentration of 1 × 10^4^ cells/well in DMEM medium with 1X antibiotic antimycotic solution and 10% fetal bovine serum (HiMedia, India) in a CO_2_ incubator at 37°C with 5% CO_2_. The cells were rinsed with 200 *μ*l of 1X PBS before being cultured for 24 h with various test concentrations of the chemical in a serum-free medium. After the treatment period, the media was aspirated from the cells. In a CO_2_ incubator, 0.5 mg/ml MTT prepared in 1X PBS was added and incubated at 37°C for 4 h. Following the incubation time, the MTT-containing media was removed from the cells and washed with 200 *μ*l of PBS. The produced crystals were properly mixed after being dissolved in 100 *μ*l of DMSO. The formazan dye turns purple-blue, and the absorbance was measured at 570 nm with a microplate reader.

## 3. Results and Discussion

### 3.1. Spectral Characterization

The electronic spectra of the complexes were recorded in acetonitrile solution. Both the complexes exhibited three bands. The spectrum showed a strong absorption band at 239, 279 nm and 241, 304 nm for complexes 1 and 2, respectively (Figures S1 and S2). These bands are assigned to *π* − *π*^*∗*^ intraligand transitions of the aromatic ring. The absorption bands at 382 and 371 nm were attributed to the presence of peroxo to vanadium (O₂^2−^⟶V) ligand to metal charge transfer (LMCT) transition [52]. The spectrum was featureless beyond 400 nm attributed to its d^0^ electronic configuration.

In the free ligand 5-nitro-2-furoic acid, (O-H) stretching was observed at 3147 cm^−1^ and the stretching frequencies corresponding to (C=O) appear at 1680 cm^−1^ (Figure S3). In complex **1**, the absence of (O-H) stretching frequency confirms the complex formation due to the deprotonation of the (O-H) group. The (V=O) stretching is observed at 947 cm^−1^ and the (O-O) at 819 cm^−1^. In complex **1**, the (COO^−^) asymmetric and symmetric stretching frequencies were observed at 1598 cm^−1^ and 1436 cm^−1^ [53–55]. The (M-N) and (M-O) stretching occur at 433 and 524 cm^−1^ (Figure S4).

In the free ligand oxine, stretching frequencies corresponding to (O-H) and (C-N) appear at 3045 cm^−1^ and 1496 cm^−1^, respectively. Similar to complex **1**, the complex **2** also shows the following characteristic stretching frequencies, i.e., the disappearance of (O-H), (V=O): 937 cm^−1^; (O-O) and (C-O): 837 cm^−1^ and 1282 cm^−1^; (C-N):1463 cm^−1^; and (M-N) and (M-O): 484 and 540 cm^−1^ (Figure S5) [56–58].

The ^1^H and ^13^C NMR spectra of complexes **1** and **2** were recorded in d_6_-DMSO solvent. In the ^1^H NMR spectra of complexes **1** and **2**, the observed chemical shift for the aromatic proton occurs between *δ* 7.4–8.6 ppm and *δ* 7.0–8.8 ppm, respectively. In ^13^ C NMR spectra of complex **1**, the carbonyl carbon appears at 155.62 ppm (Figures S6–S9).

The mass spectra of complexes **1** and **2** show their molecular ion peaks (M^+^) at 411 *m*/*z* and 417.06 *m*/*z*, respectively. The spectra confirm the molecular formula [C_15_H_10_N_3_O_8_V] and [C_19_H_16_N_3_O_5_V] for the two complexes (Figures S10 and S11).

### 3.2. X-Ray Crystallography

**Figure 1:**
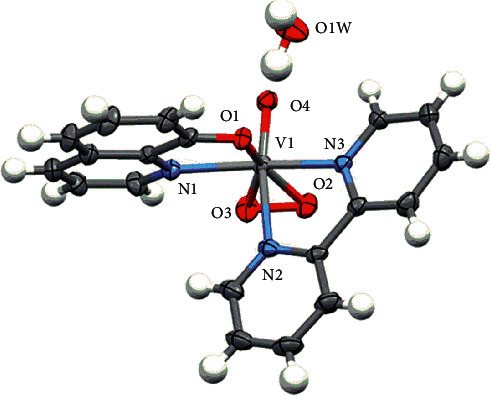
MERCURY drawing of complex **2**. Thermal ellipsoids drawn at 30% probability level.

The MERCURY drawing of complex **2** is shown in Figure 1. The structure, bond distances, and angles are given in Tables 1–3, respectively. The mononuclear structure of the vanadium atom was surrounded by the two bidentate (N, N) (N, O) ligands such as bipyridine and oxine. The vanadium atom is coordinated to two oxygen atoms (O2 and O3) from the peroxo group and two bidentate ligands (N2, N3) and (O1, N1) of bipyridine and oxine ligand, respectively. The geometry of the complex is distorted pentagonal-bipyramidal. The crystal system of complex **2** is triclinic and the space group is *P*-1. The unit cell dimensions are as follows: *a* = 8.0268 (2) Å, *α* = 99.528 (1)°, *b* = 8.5512 (2) Å, *β* = 94.738 (1)°, *c* = 13.2344 (4) Å, *γ* = 96.148 (1)°. The oxovanadium (V) complex double bond V1–O4 bond length is 1.5968 (16) Å and the peroxo group V1–O2 and V1–O3 bond lengths are 1.8924 (15) and 1.8674 (16). V1–O1 and V1–N1 bond lengths are 2.0414 (14) and 2.1414 (18), respectively. The bond angles O (3)-V (1)-O (2) and O (4)-V (1)-O (1) are 44.41 (7), and 94.40 (7) ,respectively. These compounds' crystallographic data have been deposited with the Cambridge Crystallographic Data Centre (CCDC) as supplementary publication number of complex **2** is CCDC 1950180.

### 3.3. DNA Binding Study

#### 3.3.1. Electronic Absorption Spectroscopy

**Figure 2:**
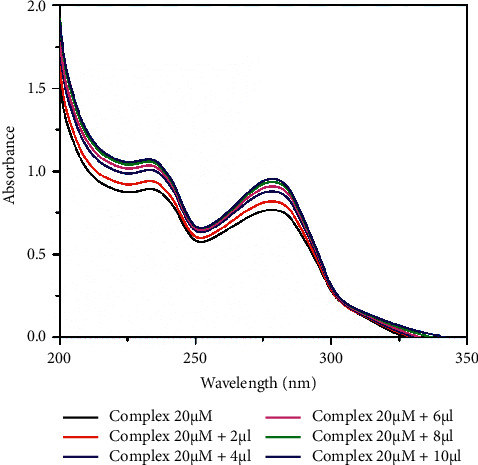
UV-absorption titration of complex **1** (20 *µ*M) with 0.2  mM CT-DNA in 10  mM Tris-HCl buffer (pH 7.2).

**Figure 3:**
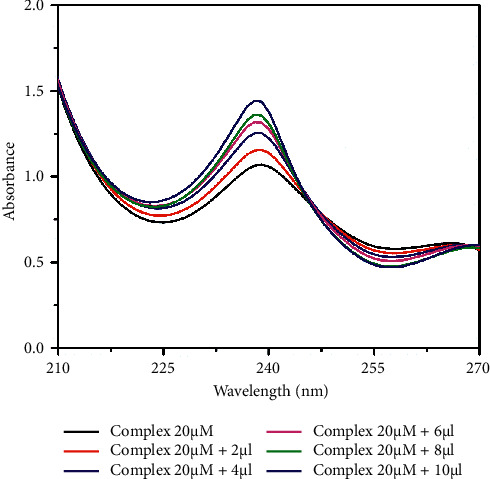
UV-absorption titration of complex **2** (20 *µ*M) with 0.2  mM CT-DNA in 10  mM Tris-HCl buffer (pH 7.2).

The electronic absorption spectral method is one of the best techniques for DNA binding studies [59, 60]. The absorption spectra of the complexes in the presence and absence of the CT-DNA were recorded in Tris-HCl buffer (pH 7.2). The peroxocomplex **1** has shown two broad absorption bands at 235 and 278 nm, and complex **2** shows bands at 238 nm, assigned to the *π* − *π*^*∗*^ intraligand transitions. In general, any changes in the double helix structure of DNA on binding to complexes are correlated to hyperchromic or hypochromic effects. The complex concentration (20 *µ*M) is kept constant and the concentration of DNA is varied. Upon the incremental addition of DNA concentration (0–10 *µ*l) to the complexes, the intensity of absorbance increases showing a hyperchromic shift due to the interaction between the DNA base pairs and aromatic chromophore of vanadium complexes. The results reveal that the groove binding mode was observed for both the complexes (Figures 2 and 3). Besides, the spectrum for complex **2** has shown the isosbestic point at 246 nm, implying that the complex bound to DNA is homogeneous [61, 62].

**Figure 4:**
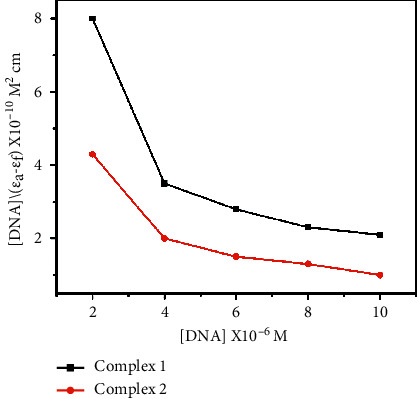
Plots of (DNA)/(*εa*−*εf*) vs. (DNA).

The intrinsic DNA binding constant (*K*_*b*_) is calculated using the following equation:

(3) $$\begin{array}{c} \frac{\left[\text{DNA}\right]}{\varepsilon_{a}-\varepsilon f}=\frac{\left[\text{DNA}\right]}{\left(\varepsilon_{b}-\varepsilon_{f}\right)}+\frac{1}{K_{b}\left[\left(\varepsilon_{a}-\varepsilon f\right)\right]}, \end{array}$$

where (DNA) is the concentration of DNA in the base pairs, the absorption coefficients *ε*_*a*_, *ε*_*f*_, and *ε*_*b*_ correspond to A_obsd_/[M], the extinction coefficient of the free compound, and the extinction coefficient of the compound when bound to the DNA, respectively. On plotting the values of (DNA)/(*ε*_*a*_*−ε*_*f*_) vs. (DNA), the *K*_*b*_ value is given by the ratio of slope to the intercept (Figure 4). The binding constants (*K*_*b*_) of complexes **1** and **2** are 8.7 × 10^3^ M^−1^ and 8.6 × 10^3^ M^−1^, respectively, and thus, complex **1** shows better binding affinity than complex **2**.

#### 3.3.2. Fluorescence Studies of Competitive Displacement Assay with Ethidium Bromide (EtBr)

**Figure 5:**
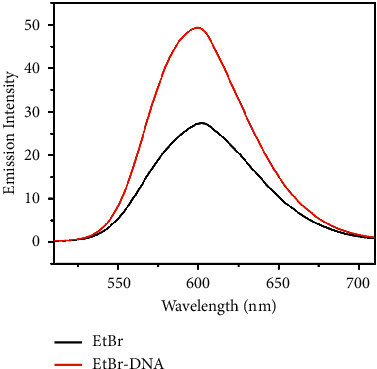
Fluorescence spectra of EtBr alone and EtBr-DNA.

**Figure 6:**
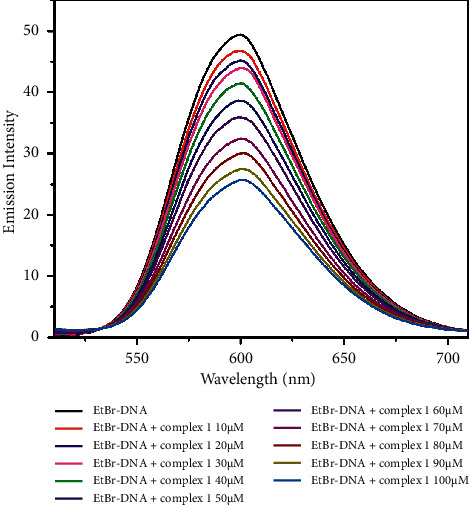
Fluorescence spectra of EtBr bound to DNA with increasing amounts of complex **1** (0–100 *µ*M).

**Figure 7:**
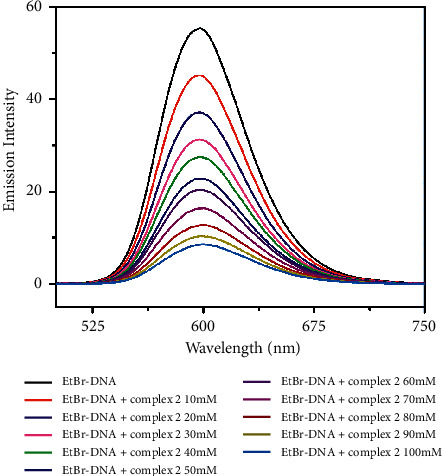
Fluorescence spectra of EtBr bound to DNA with increasing amounts of complex **2** (0–100 *µ*M).

The binding affinity of the complexes was examined using the fluorescence quenching method. Ethidium bromide (EtBr) is an organic cationic dye, a well-known DNA intercalator, and acts as a fluorescent tag. The displacement assay was done using EtBr bound CT-DNA solution (EtBr = 10 *µ*M and DNA = 100 *µ*M) acting as a probe. The EtBr emission intensity was increased, when it was intercalated into DNA base pairs and the emission wavelength was 601 nm (Figure 5). In addition, by increasing the concentration of complexes (0–100 *µ*M) gradually to EtBr-DNA, the fluorescent intensity decreased due to the displacement of EtBr by complexes [63–65]. The quantum yield of complexes **1** and **2** was 1.2 and 1.5, respectively. The fluorescence spectra are shown in Figures 6 and 7. The extent of binding is quantified by calculating the Stern–Volmer constant, *K*_SV_.

**Figure 8:**
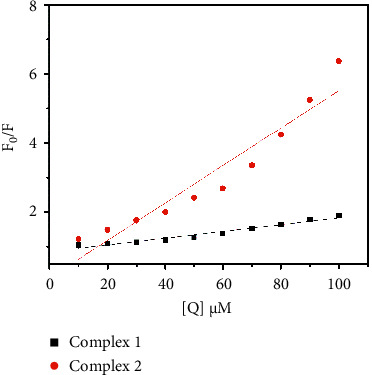
Stern–Volmer plots of complexes **1** and **2**.

The linear Stern–Volmer equation is

(4) $$\begin{array}{c} \frac{F_{0}}{F}=1+K_{\text{SV}}\left[\text{DNA}\right], \end{array}$$

where *F* and *F*_0_ are the emission intensities in the presence and absence of the quencher and K_SV_ is the Stern–Volmer constant. The *K*sv is calculated from the slope of the plot *F*_0_/*F* versus *Q* (Figure 8). The *K*_SV_ value of complexes **1** and **2** are 9.7 × 10^3^ M^−1^ and 5.4 × 10^4^ M^−1^, respectively.

#### 3.3.3. Viscosity Study

**Figure 9:**
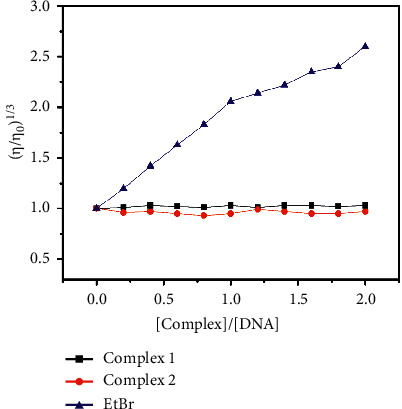
Effect of increasing amounts of EtBr and complexes **1** and **2** (20–200 *µ*M) on the relative viscosities of CT-DNA (100 *µ*M) in Tris-HCl buffer at room temperature.

The viscosity experiment is one of the effective methods to determine the binding mode between the DNA and complexes. Small molecules interact with DNA, and the intercalative binding mode changes the DNA conformation resulting in increasing the length of DNA and thereby increasing the relative viscosity of DNA. In contrast, electrostatic and groove binding modes would rather show no significant effect or less change in DNA viscosity [66, 67]. Ethidium bromide (EtBr) with DNA and buffer shows a marked increase in viscosity inferring intercalative binding mode; whereas, complexes **1** and **2** show only marginal changes in viscosity with an increase in concentration suggesting the complexes bind with DNA through groove binding mode (Figure 9).

### 3.4. DNA Cleavage

#### 3.4.1. Gel Electrophoresis Method

**Figure 10:**
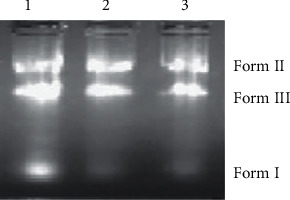
Cleavage pattern of pBR322 DNA by complexes **1** and **2**. Lane 1, control; lane 2, complex **1**+ DNA; lane 3, complex **2**+ DNA.

The pBR322 plasmid DNA cleavage activity of peroxovanadium complexes was studied by using the agarose gel electrophoresis method. The molecules migrate in the gel as a function of their mass, charge, and shape. DNA cleavage shows supercoiled circular (form I) changed into nicked circular (form II) and linear (form III) [68–70]. The fastest migration was observed for form I and the slowest migration for form II, and the pace of migration for form III was in between form I and form II. The results are shown with supercoiled circular, nicked, and linear forms without adding light or reducing agents. The complexes bring about pBR322 DNA cleavage hydrolytically. The gel picture of the complexes containing the bands is shown in Figure 10.

### 3.5. BSA Binding Study

#### 3.5.1. Fluorescence Quenching Studies

**Figure 11:**
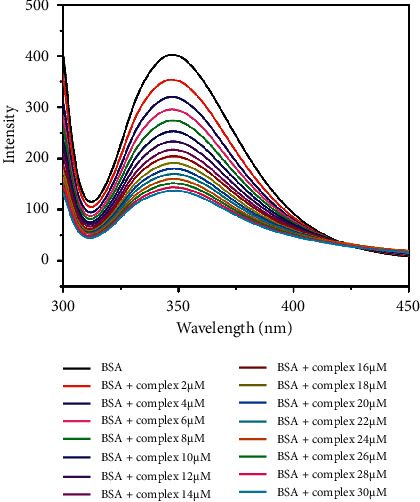
Fluorescence spectra of BSA (2 *μ*M) with complex **1** (0–30 *μ*M) in PBS buffer (pH 7.4).

**Figure 12:**
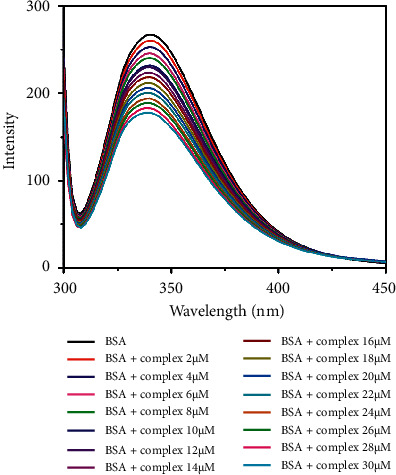
Fluorescence spectra of BSA (2 *μ*M) with complex **2** (0–30 *μ*M) in PBS buffer (pH 7.4).

Protein is one of the primary molecular targets of anticancer medicines. Fluorescence spectroscopy is the best technique to evaluate the interaction between BSA and metal complexes. The protein binding ability of complex can be studied using tryptophan fluorescence quenching experiments using BSA as the substrate in PBS (phosphate-buffered saline) (pH 7.4). Protein contains three aromatic amino acid residues such as tryptophan, tyrosine, and phenylalanine, but the fluorescence of BSA arises mainly due to two tryptophan residues, Trp-134 and Trp-212. Trp-212 is located within a hydrophobic binding pocket in subdomain IIA and Trp-134 is located on the surface of subdomain IB. The absorption and fluorescence emission wavelength maxima are observed at 280 nm and 345 nm, respectively. The fluorescence spectra of BSA were recorded in the absence and the presence of increasing concentrations of complexes. The fluorescence intensity of the protein, observed at around 345 nm, decreases as the complex concentration increases without any shifts towards lower or higher wavelengths [71] (0–30 *µ*M) (Figures 11 and 12). This indicates that there is no alteration in the local dielectric environment of BSA, which would otherwise cause shifts in emission maxima. This quenching effect may be caused by subunit associations, protein conformational transitions, denaturation, or substrate binding.

**Figure 13:**
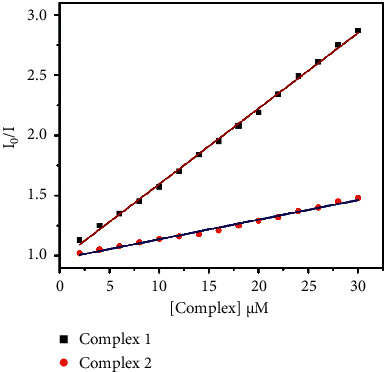
Stern–Volmer plots of *I*_0_/*I* vs. (complex).

The Stern–Volmer equation is used to calculate the quenching constant (*K*_BSA_). Stern–Volmer graphs of *I*_0_/*I* vs. (complex) (Figure 13) are created using corrected fluorescence data that takes dilution into account. The equation can be used to create a linear fit of the data.

(5) $$\begin{array}{c} \frac{I_{0}}{I}=1+K_{\text{BSA}}\left(Q\right)=1+kq\tau_{0}\left(Q\right), \end{array}$$

where *I* and *I*_0_ are the emission intensities of BSA in the presence and absence of a quencher of concentration (*Q*), respectively, which gave the quenching constant (*K*_BSA_) using Origin Pro 8.5 software. *τ*_0_ is the average lifetime of the tryptophan in BSA without quencher reported as 1 × 10^−8^s, and *k*_*q*_ is the quenching rate constant.

The mechanism of fluorescence quenching is classified into different types such as static quenching, dynamic quenching, and combined static and dynamic quenching. The formation of a fluorophore quencher complex is a part of the static quenching process. Dynamic quenching refers to the mechanism through which the quencher and fluorophore come into contact during the transient existence of the excited state. The *k*_*q*_ quenching rate constants of the complexes **1** and **2**, 6.28 M^−1^S^−1^ and 1.6 M^−1^S^−1^, are higher than the maximum scattering collision quenching constant, 2 × 10^10^ M^−1^S^−1^, suggesting a static fluorescence quenching mechanism.

The binding propensity of the quenchers with respective serum proteins is expressed by the Scatchard equation[72, 73]:

(6) $$\begin{array}{c} \frac{\text{log}\left(I_{\text{0}}-I\right)}{I}=\log\;K+n\text{log}\left(Q\right). \end{array}$$

For such static quenching interaction, the binding constant (*K*) and the number of binding sites (*n*) can be determined.

**Figure 14:**
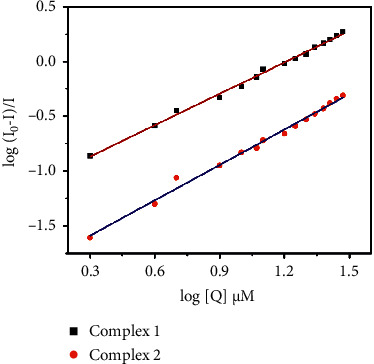
Scatchard plots of log (*I*_0_−*I*)/*I* vs. log (*Q*).

The linear fitting of the log (*I*_0_−*I*)/*I* vs. log (*Q*) plot gives the values of *n* and *K* from the slope and the intercept (Figure 14). The quenching constants are 0.0628 × 10^6^ M^−1^ and 0.0163 × 10^6^ M^−1^; quenching rate constants are 6.28 × 10^12^ M^−1^S^−1^ and 1.6 × 10^12^ M^−1^S^−1^ for complexes **1** and **2**, respectively. The binding constants (*K*) and binding sites (*n*) are 0.06 × 10^6^ M^−1^ and 0.9 for complex **1** and 0.01 × 10^6^ M^−1^ and 1 for complex **2**. The value *n* indicates the binding sites for the complex on the BSA molecule. The binding constant of complex **1** shows a greater value than complex **2** (Table 4).

#### 3.5.2. UV-Visible Absorption Studies

**Figure 15:**
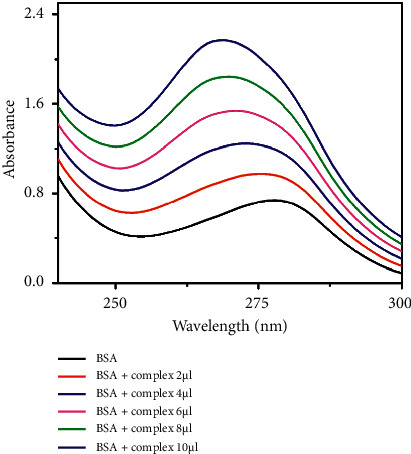
UV absorption titration of complex **1** (0–10 *µ*l) with BSA (1 *µ*M) in PBS buffer (pH 7.4).

**Figure 16:**
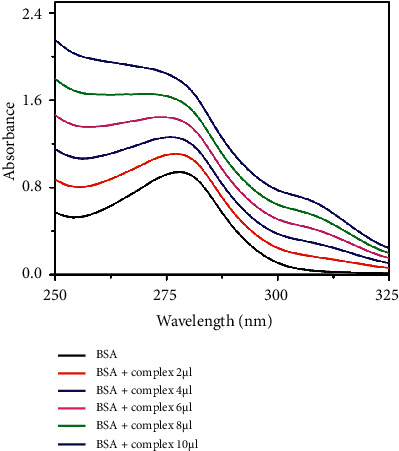
UV absorption titration of complex **2** (0–10 *µ*l) with BSA (1 *µ*M) in PBS buffer (pH 7.4).

UV-Vis absorption method is a simple method to examine the possible quenching mechanisms. The spectrum was recorded in the absence and the presence of the increasing concentration of the vanadium complexes (Figures 15 and 16). The spectrum of BSA shows a band at 279 nm, due to the aromatic amino acid residues (Trp, Tyr, and Phe). Upon the addition of the complex concentration (0–10 *µ*l) gradually to BSA, the absorbance value increases with a blue shift confirming the interaction between BSA and vanadium complexes. The results reconfirm the static quenching mechanism of the complexes, forming a complex-BSA in the ground state [74–76].

### 3.6. DFT Study

**Figure 17:**
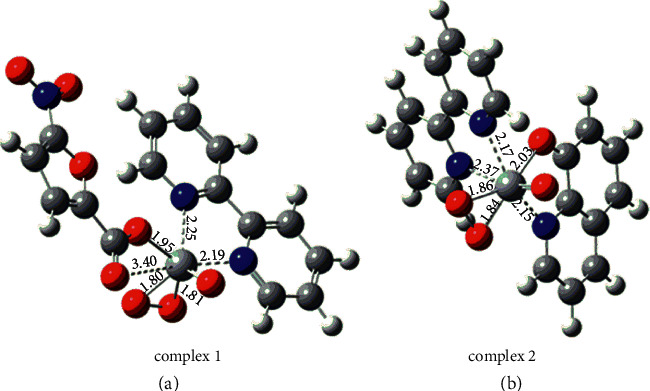
Optimized geometry of complexes (a) 1 and (b) 2 obtained at wb97xd/6–311++g (2d, 2p) level of theory.

The optimized geometry of complexes **1** and **2** was obtained at wb97xd/6–311++g (2d, 2p) level of theory as shown in Figure 17. The coordination bonding pattern and other geometrical parameters are depicted and are comparable with experimental observations (Table 5). The vanadium atom is coordinated with seven atoms and the geometry of the complex is distorted pentagonal-bipyramidal. The calculated bond lengths of V (1)–O (1), V (1)–O (2), and V (1)–O (3) are 2.03, 1.84, and 1.86, respectively. There are only a few minor differences in the structural features of the complexes when the data obtained from DFT calculations and X-ray crystallography are compared [77].

**Figure 18:**
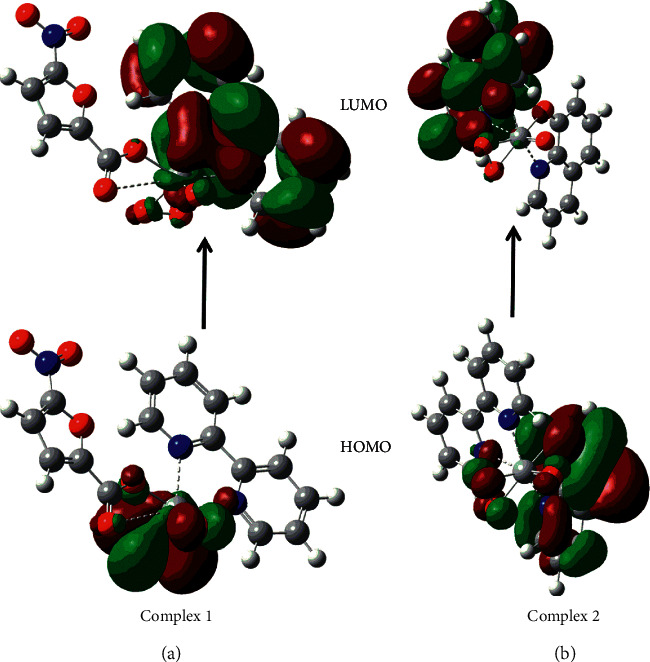
Frontier molecular orbitals density plots of complexes 1 (a) and 2 (b) obtained at wb97xd/6–311++g (2d, 2p) level of theory.

The frontier molecular orbitals are the highest occupied molecular orbital (HOMO) associated with electron-donating potential and the lowest unoccupied molecular orbital (LUMO) related to electron affinity [78, 79]. The frontier molecular orbital density plots shown in Figure 18 would dictate the reactivity and stability of metal complexes. In complex **1**, HOMO is distributed over the oxygen atom and LUMO is distributed over the bipyridine molecule. In complex **2**, HOMO is distributed over the oxine molecule and oxygen atom and LUMO is distributed over the bipyridine molecule. The *E*_HOMO-LUMO_ energy gaps of complexes **1** and **2** are calculated to be 0.253 eV and 0.242 eV, respectively.

### 3.7. Molecular Docking Studies on Metal Complexes Binding to E2 Binding Region of DNA

**Figure 19:**
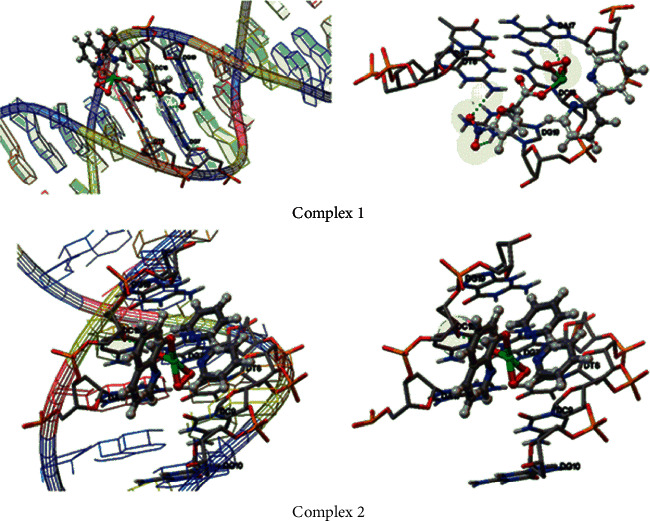
Docked conformations of the complexes complex **1** (top) and complex **2** (bottom) with the crystal structure of the E2 binding region of DNA (PDB ID: 423D). The compounds are represented in the ball and stick model. The green dotted line represents the hydrogen bonds between the metal complex and DNA.

Molecular docking calculations are carried out with both the major groove and the minor groove as binding sites for the metal complexes. It has been observed that the complexes bind to the major groove of double-stranded DNA. The binding (∆G_BE_) and intermolecular energies (∆G_intermol_) of complexes **1** and **2** are given in Table 6. The best-docked conformations of complexes **1** and **2** obtained by docking calculations are shown in Figure 19. The docked complexes of **1** and **2** possess binding energy in the range of−7.35 and−7.0 kcal/mol. This is due to the strong contribution of intermolecular van der Waals, hydrogen bonding, and desolvation energies. It can also be observed that complex **1** forms three explicit hydrogen bonds with DG7, DA17, and DG19 residues, while complex **2** has only one hydrogen bond with DC18 residue which could be the reason for the stronger interaction observed in the case of complex **1** over complex **2**.

The binding ability of complexes to DNA is investigated through molecular docking procedures. Here, the E2 regulatory protein binding DNA targets (PDB ID: 423D) are considered for the calculations [80, 81]. The regulatory signals of the virus are dependent on the nucleotides that are involved in protein binding along with the deformation ability of the corresponding target DNA region. Therefore, it is proposed that blocking the above region through the introduction of inhibitory metal complexes that can strongly bind to the key oligonucleotides can consequently inhibit the regulatory signals of the DNA. The binding energy of complex **1** is greater as shown by its high binding constant value of 8.7 × 10^3^ M^−1^.

### 3.8. In Vitro Cytotoxic Activity

**Figure 20:**
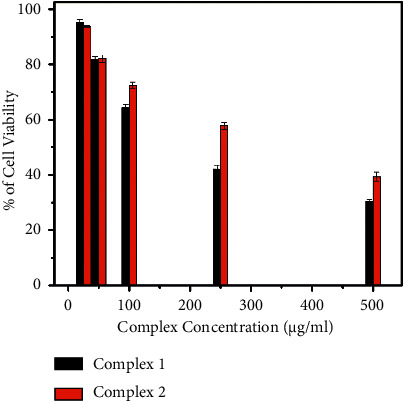
Cell viability of complexes **1** and **2**.

**Figure 21:**
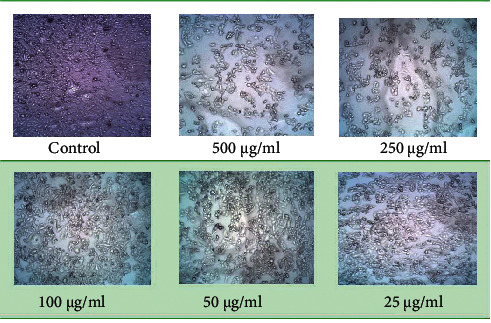
Cell viability images of complex **1**.

The cytotoxicity of complexes **1** and **2** was tested against the HeLa cell line by using the MTT assay method. Cisplatin cytotoxic activity is the standard reference for the comparison purpose and the IC_50_ value is 24 ± 1.46 *µ*M. The plot of the percentage of the cell viability versus complex concentration is shown in Figure 20. The cell viability decreases with increased complex concentration indicating a dose-dependent growth inhibitory effect (Figures 21 and S12). Furthermore, the IC_50_ value for complexes **1** and **2** against the HeLa cell line is calculated and is found to be 512 ± 4.27 *µ*M and 788 ± 26.57 *µ*M, respectively, showing a moderate cytotoxic effect. From the result, cisplatin shows higher cytotoxic activity, but the vanadium complexes exhibit a lower cytotoxic effect. Peroxovanadate complexes exert their probable mode of anticancer action through the inhibition of protein tyrosine phosphatase, lipoperoxidation, DNA cleavage, and strand breakage associated with ROS and act as potential cytotoxic agents [10].

## 4. Conclusion

The oxoperoxovanadium (V) complexes have been synthesized and characterized by spectral techniques and single-crystal X-ray diffraction studies. The crystal system of complex **2** is triclinic and the geometry is found to be distorted pentagonal-bipyramidal. The complexes have shown groove binding mode with DNA and the binding constant values are assessed and supported by a molecular docking study. The strong binding interactions with BSA are evaluated, and the complexes have shown a moderate cytotoxic effect on HeLa cell lines. Based on the results obtained, it is observed that complex **1** shows stronger DNA/BSA binding ability than complex **2**. Further studies are required to assess the probable mechanism through which the complexes exert their cytotoxic activity and are underway.

## Figures and Tables

**Table 1:** Crystal data of complex **2**.

Empirical formula	C_19_H_16_N_3_O_5_V
Formula weight	417.29
Temperature	100 K
Wavelength	1.54178
Crystal system	Triclinic
Space group	P-1

Unit cell dimensions	*a* = 8.0268 (2) Å
	*α* = 99.528 (1)°
	*b* = 8.5512 (2) Å
	*β* = 94.738 (1)°
	*c* = 13.2344 (4) Å
	*γ* = 96.148 (1)°

Volume	886.03 (4) Å^3^
Z	2
Density (calculated)	1.564 g/cm^3^
F (000)	428.0
Theta	79.072°.
Index ranges	*h* = 10, *k* = 10, *l* = 16
Reflections collected	48492
Independent reflections	(*R* (int) = 0.0377)
Final *R* indices	*R*1 = 0.0378 (3317); w*R*2 = 0.0992 (3769)

**Table 2:** Bond distances of complex **2**.

Bond length	Complex **2** (Å)
V (1)–O (1)	2.0414 (14)
V (1)–O (2)	1.8924 (15)
V (1)–O (3)	1.8674 (16)
O (2)–O (3)	1.421 (2)
V (1)–O (4)	1.5968 (16)
V (1)–N (1)	2.1414 (18)
V (1)–N (2)	2.2911 (19)
V (1)–N (3)	2.1346 (17)

**Table 3:** Bond angles of complex **2**.

Bond angle	Complex **2** (°)
O (4)-V (1)-O (3)	103.75 (9)
O (4)-V (1)-O (2)	101.08 (8)
O (3)-V (1)-O (2)	44.41 (7)
O (4)-V (1)-O (1)	94.40 (7)
O (3)-V (1)-O (1)	151.15 (7)
O (2)-V (1)-O (1)	152.48 (7)
O (4)-V (1)-N (3)	94.76 (7)
O (3)-V (1)-N (3)	122.50 (7)
O (2)-V (1)-N (3)	78.98 (7)
O (1)-V (1)-N (3)	77.16 (6)
O (4)-V (1)-N (1)	98.17 (7)
O (3)-V (1)-N (1)	78.93 (7)
O (2)-V (1)-N (1)	122.92 (7)
O (1)-V (1)-N (1)	76.41 (6)
N (3)-V (1)-N (1)	151.32 (7)
O (4)-V (1)-N (2)	167.16 (8)
O (3)-V (1)-N (2)	87.02 (8)
O (2)-V (1)-N (2)	81.61 (7)
O (1)-V (1)-N (2)	78.57 (6)
N (3)-V (1)-N (2)	73.30 (7)
N (1)-V (1)-N (2)	90.66 (7)

**Table 4:** *K*_SV_, *Kq*, (*n*), and *K* values of complexes **1** and **2**.

Compound	(*K*_SV_) *×* 10^6^ M^−1^	(*K*_*q*_) *×* 10^12^ M^−1^S^−1^	(*K*) *×* 10^6^ M^−1^	(*n*)
Complex **1**	0.0628	6.28	0.06	0.9
Complex **2**	0.0163	1.6	0.01	1

**Table 5:** Comparison between experimental and calculated bond lengths for complex **2**.

Bonds	Experimental (X-ray crystallography)	Calculated (density functional theory)
V (1)–O (1)	2.0414 (14)	2.03
V (1)–O (2)	1.8924 (15)	1.84
V (1)–O (3)	1.8674 (16)	1.86
V (1)–N (1)	2.1414 (18)	2.15
V (1)–N (2)	2.2911 (19)	2.37
V (1)–N (3)	2.1346 (17)	2.17

**Table 6:** The binding (∆G_BE_) and intermolecular energies (∆G_intermol_) of complexes **1** and **2** with DNA.

Parameter (kcal/mol)	Complex **1**	Complex **2**
Binding energy	−7.35	−7.00
Intermolecular energy	−7.65	−7.90
Vdw_hd	−7.51	−6.81
Electrostatic energy	−0.31	−1.09
Total internal energy	−0.61	−0.94
Torsional energy	+0.30	+0.89

All the energies are reported in kcal/mol.

## Data Availability

The data on Figures S1-S12, UV-Visible, FT-IR, NMR, Mass spectra of the complexes supporting data are included within the supplementary materials.

## Supplementary Materials

Supplementary Materials Figures S1-S12, UV-visible, FT-IR, NMR, and mass spectra of the complexes supporting data are included in the supplementary information files.
