# Supplementary material for: Oxoperoxovanadium Complexes of Hetero Ligands: X-Ray Crystal Structure, Density Functional Theory, and Investigations on DNA/BSA Interactions, Cytotoxic, and Molecular Docking Studies
Source: Bioinorg Chem Appl. 2022 Aug 17;2022:8696420. doi: 10.1155/2022/8696420 (PMC9402336; doi:10.1155/2022/8696420)
Supplement: Supplementary Materials — Figures S1-S12, UV-visible, FT-IR, NMR, and mass spectra of the complexes supporting data are included in the supplementary information files. [file 8696420.f1.docx]

Figure S1: UV- Visible spectrum of Complex 1 (2.4 X 10^-5^ M) in acetonitrile





Figure S2: UV-Visible spectrum of Complex 2 (2.4 X 10^-5^ M) in acetonitrile

Figure S3: FT-IR spectra of 5Nitro 2 furoic acid

Figure S4: FT-IR spectra of complex 1


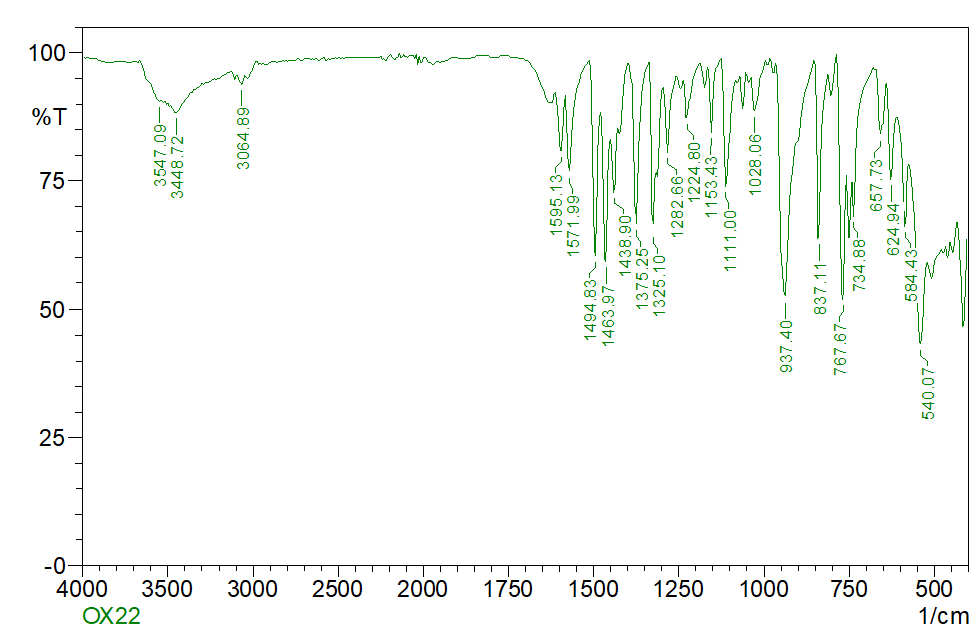


Figure S5: FT-IR spectra of complex 2


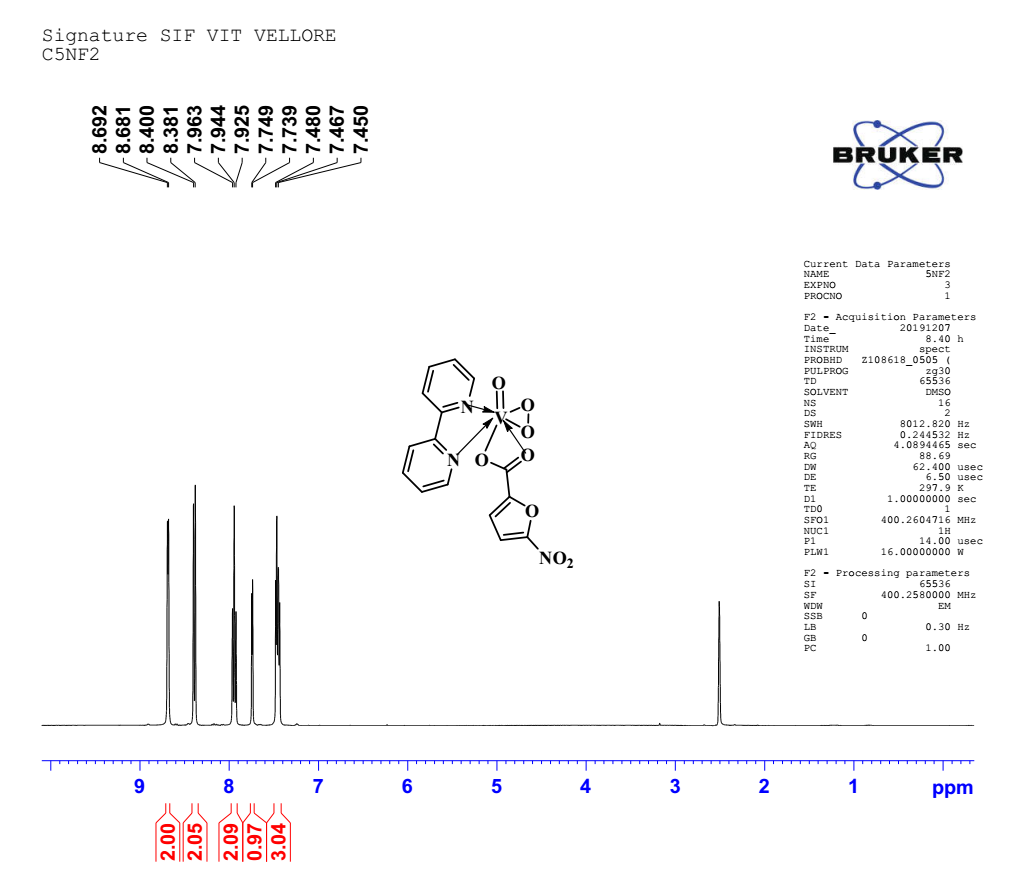


Figure S6: ^1^H NMR of complex 1


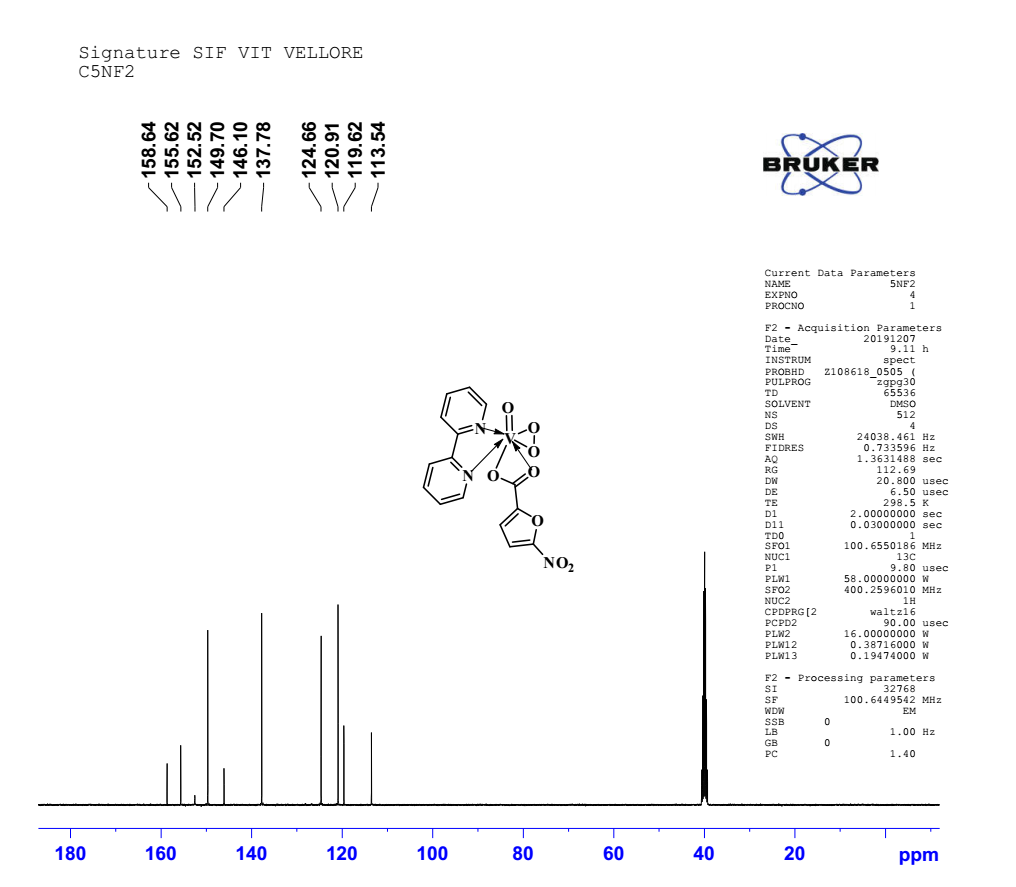


Figure S7: ^13^C of complex 1


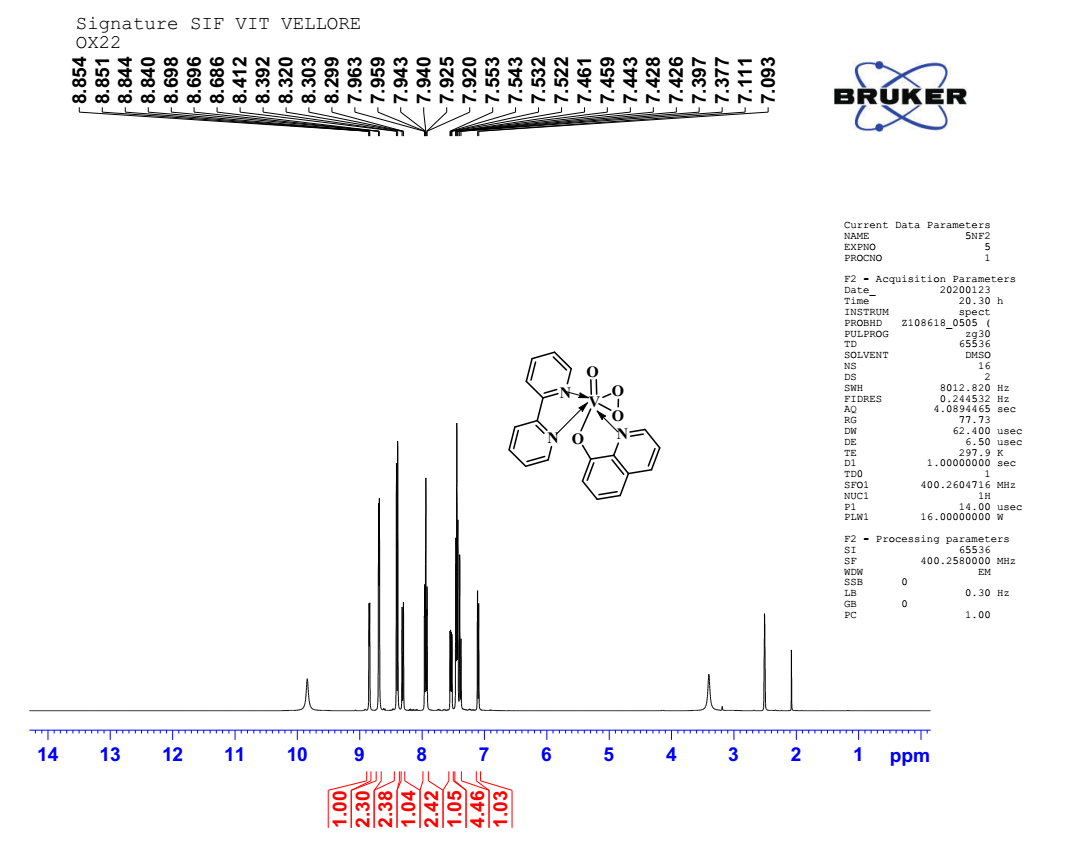


Figure S8: ^1^H NMR of complex 2


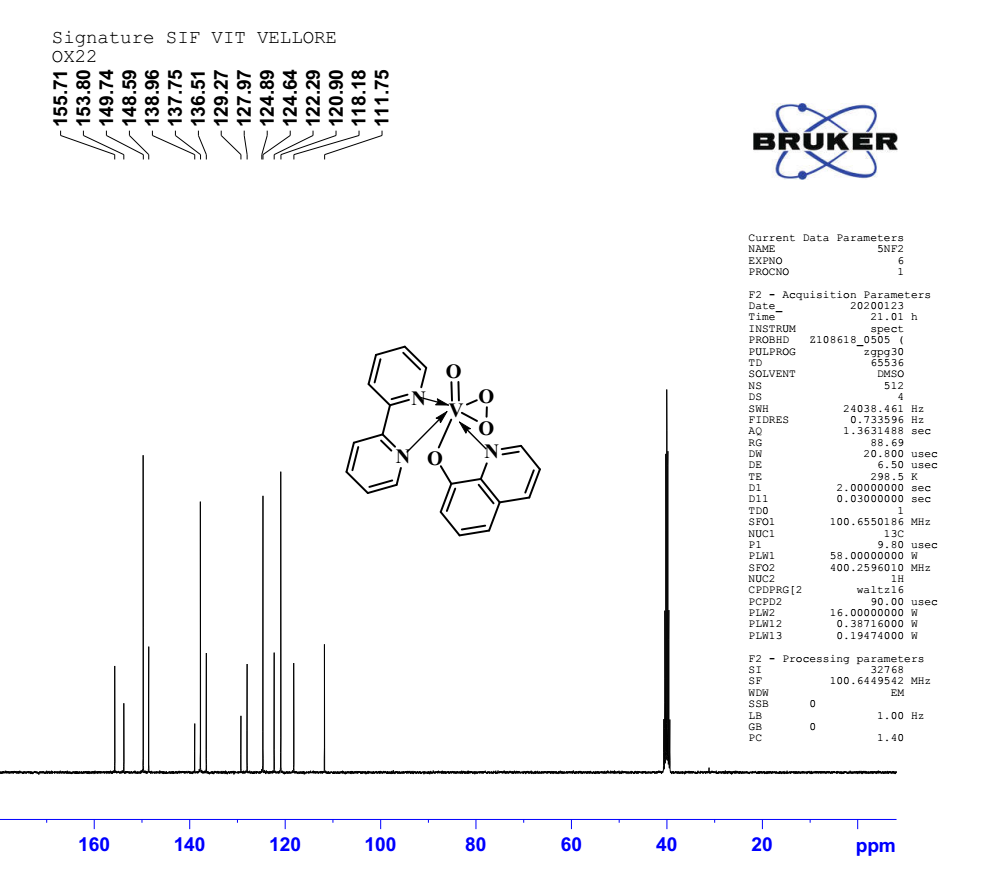


Figure S9: ^13^C NMR of complex 2


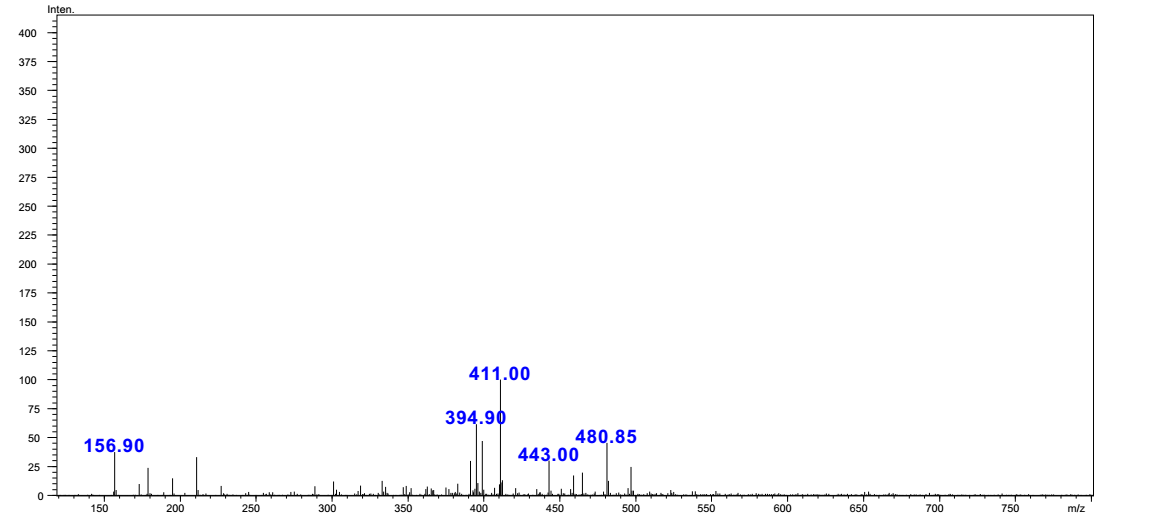


Figure S10: Mass spectra of complex 1

Figure S11: Mass spectra of complex 2


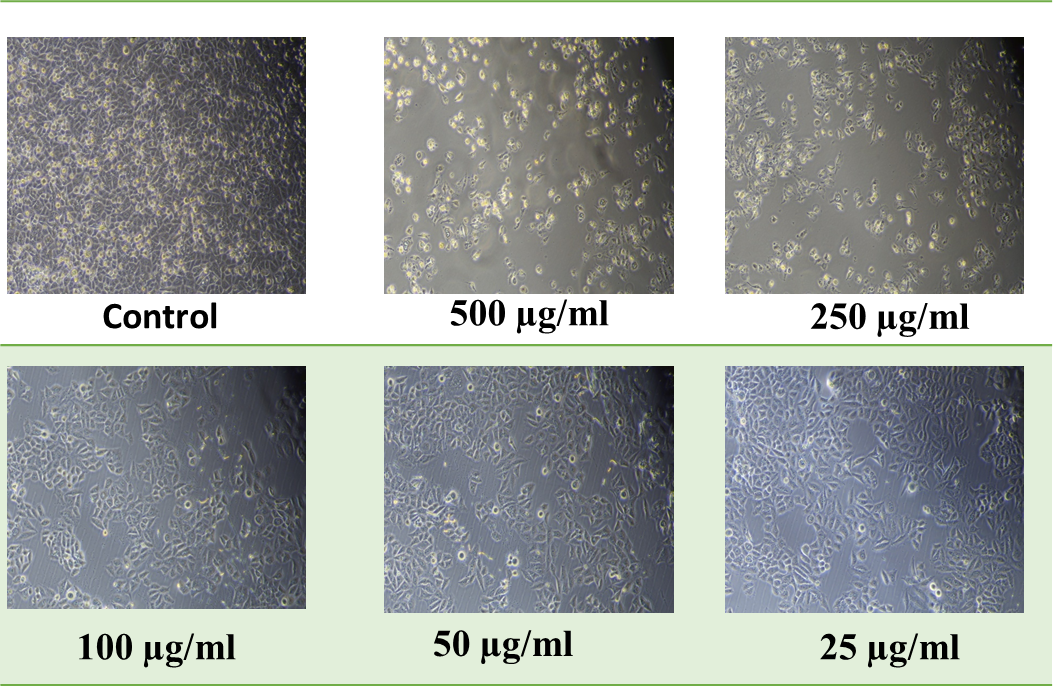


Figure S12: Cell Viability Images of Complex 2
